# Diagnosing sex: Intersex surgery and ‘sex change’ in Britain 1930–1955

**DOI:** 10.1177/1363460717740339

**Published:** 2018-01-17

**Authors:** David Andrew Griffiths

**Affiliations:** University of Surrey, UK

**Keywords:** history, intersex, medicine, surgery, trans

## Abstract

The medical ‘management’ of individuals with atypical sex characteristics, or intersex variations, has been under scrutiny since the beginnings of intersex activism in the 1990s. This article explores a history of intersex surgeries in Britain and the interaction with medical and popular discourses around ‘sex-change’ between 1930 and 1955. A focus on this period in Britain helps to critically elaborate on debates in intersex scholarship; provides historical context for the introduction of approaches and protocols based on John Money and colleagues’ work in the USA in the mid-century; and analyses a long history of tension and intersection between trans and intersex experiences, treatments, politics and popular representations that continue into the present.

## Introduction

Intersex scholarship since the 1990s has recognized the need to place contemporary practices, protocols and politics in their social and historical contexts (Fausto-Sterling, 2000; Kessler, 1990, 1998). This article contributes to debates in the historical study of intersex, as well as to present concerns in intersex politics and activism.^1^ While there are historical accounts of intersex in the 19th century that include the UK (Dreger, 1998; Mak, 2012), and investigations of the contemporary US context (Davis, 2015; Feder, 2014; Preves, 2003), there is little published historical work on medical approaches to intersex in the UK after the 1910s. Bioethicist Ellen Feder has drawn attention to this gap in knowledge, highlighting a shift from a flow of biomedical knowledge and influence from the UK to the USA in the late 19th and early 20th centuries, to a reversal of this flow in the mid-20th century. She suggests that more detailed analysis of this period would be illuminating not just for historical scholarship but also for bioethics and other fields (Feder, 2014: 214, n. 3). I respond to this gap in the literature by focusing on Britain between 1930 and 1955, a period in which so-called ‘sex change’ operations were controversial, while intersex surgeries, including the removal of healthy gonadal and genital tissue, were becoming routine.^2^

In this article I read multiple hybrid texts: texts that do the translation work between the overlapping categories of intersex and trans, including published case studies, media representations, letters, and autobiographies. I analyse how individuals within the medical profession attempted to purify these fuzzy and overlapping categories, through the separation of children and adults, intersex and trans, biology and psychology, into distinct non-overlapping ontological categories (Latour, 1993). I begin by analysing gender differences in the clinical encounter as recounted by Lennox Ross Broster, who was performing routine intersex surgeries in Britain from the 1930s until the beginning of the Second World War. This analysis brings in representations from the popular British press who reported on Broster’s work, particularly with Mark Weston, an athlete who re-registered male (after competing in the Olympics as a woman) following surgeries in 1935. Developments in biomedical science and stories in the press provided a language with which individuals could approach the medical profession to request surgeries to enable their bodies to better conform to their inner sense of sex. I will analyse two such individuals in the UK: Michael Dillon, who had a mastectomy in 1942 and began a long series of surgeries in 1946 including the first recorded phalloplasty; and Roberta Cowell, who received an orchidectomy from Dillon himself (he was a medical student) sometime before having a vaginoplasty in 1951.^3^

While Dillon and Cowell have been discussed for their relevance to histories of trans politics and experiences, I will argue that they are more relevant to the history of intersex than has been noted. As scholars have pointed out, both intersex and trans individuals have been pathologized by the medical profession’s insistence on a strict binary model of sex, gender and sexuality; this is true in the past and in the contemporary context (Davis et al., 2016; Meyerowitz, 2002). I analyse Dillon and Cowell’s appearance in case studies, in the media, and in their own autobiographies. Autobiographies may well be ‘unreliable’ historical texts in this context (Stone, 2014 [1987]). However, these non-medical accounts also demonstrate that biomedical language ‘is never alone in the field of empowering meanings’ (Haraway, 1989: 3). Dillon and Cowell represent actors in the medical network. While Dillon was a medical student and therefore somewhat part of the community of practice that is the medical profession, Cowell was an outsider. Despite this, they both had to navigate social and moral structures and hierarchies to access the medical treatments that they needed. I follow the insight from feminist science and technology studies that vision may be better from these less powerful, subjugated and always partial perspectives (Haraway, 1991; Star, 1990).

Following this, I turn to the 1950s – a significant period for the history of intersex. Following his 1952 PhD dissertation ‘Hermaphroditism: An Inquiry into the Nature of a Human Paradox’, John Money and colleagues at Johns Hopkins began to develop a set of ‘case management’ guidelines for individuals with atypical sex anatomies, or intersex variations, which had truly global impact (Money, et al., 1955a, 1955b, 1956). These guidelines suggested that, although chromosomal, gonadal, genital and hormonal markers of sex were important in the diagnosis of sex, the gender of rearing was considered the best way of predicting adult ‘gender role’, a term Money and colleagues coined in 1955. The guidelines recommended early surgical intervention on genitals that did not conform to cultural ideas of what male and female genitals should look like, and consistent rearing in one corresponding gender. Money’s mid-century work on intersex ‘constituted the essential writing on the subject until the founding of the Intersex Society of North America (ISNA) in 1993, which directly challenged their theories’ (Reis, 2009: 116–117). Some scholars have described Money’s work as a ‘radical departure’ from earlier practice and protocols (Karkazis, 2008). Others, however, have stressed that his work should not be separated from ‘trends in psychology, surgery, and intersex management’ prior to 1955 (Reis, 2009: 114), as well as existing practices in the USA (Eder, 2011). In this article, I illuminate this discussion of the importance of Money in scholarship on intersex, by bringing to light a previously unexamined history of intersex and ‘sex-change’ surgeries in the UK, prior to the influence of his guidelines.

## 1930s intersex treatments in Britain

Certain surgical interventions on intersex bodies were routine in the UK in the 1930s. Lennox Ross Broster, made full surgeon in 1933 at Charing Cross Hospital in London, was a prominent British practitioner of these interventions (Obituary Notices: L R Broster, 1965). He mainly saw adult women with what was known at the time as the ‘adreno-genital syndrome’, a term no longer in use but bearing a resemblance to the contemporary diagnosis of Congenital Adrenal Hyperplasia (CAH). For Broster, the adreno-genital syndrome involved the over-activity of the adrenal gland, and a range of physical differences including a larger than average clitoris that may look similar to a penis, labia that resemble a scrotum, and differences in distribution of body hair and body fat. In 1932, Broster and colleagues published an article on a new technique that allowed for the surgical removal of the adrenal glands in these cases, a procedure he used throughout the 1930s (Broster et al., 1932). In 1933, he published *The Adrenal Cortex,* in which he presented 10 case studies of ‘women with virilism’ (Broster and Vines, 1933). In 1938, he published *The Adrenal Cortex and Intersexuality* in which he claimed to have seen over 100 patients and presented a wealth of case studies (Broster et al., 1938). However, as I will explain later in this article, Broster saw individuals with a range of bodily variations, not just those with the ‘adreno-genital syndrome’.

Broster was an endocrinologist, but claimed in 1938 that ‘[p]erhaps the most interesting, disquieting, and to the clinician the most perplexing, feature of these patients, is their psychological outlook’, despite the 1933 volume not addressing this subject (Broster et al., 1938: 6). Broster claimed that adrenalectomy was an efficient tool for normalizing both biology and psychology:

As a result of operative treatment it has been discovered that these patients show not only a general and immediate tendency to lose their acquired male characters, and revert to their normal feminine ones, but also to return to normal sexuality psychologically, when this has been abnormal before operation. (Broster et al., 1938: 5)

On psychological aspects, he worked with Clifford Allen, who was the consultant psychiatrist at Charing Cross, the Physician in Charge of the Psychiatric Department of the Seamen’s Hospital, Greenwich, and the Assistant Physician to the Institute of Medical Psychology at the Tavistock Clinic in Bloomsbury. Broster and Allen shared the view that atypical biology could lead to atypical psychology; if adrenal glands were thought to masculinize the biological, then they would be expected to influence sex roles, aims and object choice as well.^4^ For both Broster and Allen, biological normality was structured into a binary of male and female bodies, and linked to a strict psychological normality, as measured by heterosexuality.

Broster largely saw adults, particularly at the start of his research, although he saw an increasing number of younger and pre-pubescent cases just prior to the interruption of his work caused by the Second World War (Broster, 1953). The published case studies narrate a consultative process between the medical professionals, the young adults who sought medical treatment, and often their parents. I will discuss three cases from *The Adrenal Cortex and Intersexuality* (Broster et al., 1938) to demonstrate that, while adult individuals were involved in the consultative process regarding their assigned sex and associated medical procedures, there was the sense of an underlying ‘true’ sex, even if that sex was not wholly defined by gonads or hormones and inseparable from contemporary understandings of male and female psychology and social roles. These examples also demonstrate that Broster was doing routine examinations and surgical interventions on a range of atypical sex anatomies, or intersex variations. Broster discusses a 16-year-old female patient who had not begun to menstruate or develop breasts. Upon examination, ‘hypospadias with a moderate-sized penis and two small undescended testes’ were found. He reports simply that ‘[o]rchidectomy was performed as she and her family wished her to remain feminine’ (Broster et al., 1938: 45).^5^ It seems from this example that adult individuals could be involved in the decision-making process around their treatment and sex-assignment. A second example, however, reveals that Broster’s personal opinion and social concerns did intrude into his case studies. In a description of a case where a woman wished to remain a woman, even after the discovery of testes, he concludes with the exclamation: ‘Since then she has adopted a son!’ (Broster et al., 1938: 45). The exclamation mark betrays a possible astonishment at the woman becoming a mother. Such comments reveal that Broster was still to some extent relying on an underlying norm of ‘true sex’ which this patient was seen as lacking; however, this ‘truth’ of sex is located in a complex interrelationship of gonads, hormones, ‘psychology’ and social roles.

A third example in *The Adrenal Cortex and Intersexuality* is a famous case and reveals a different attitude. Mark Weston was born around 1906 and competed nationally and internationally as a woman for Britain in the javelin, shot-put and discus. In 1935, he was admitted to Charing Cross Hospital where he underwent at least two surgical procedures. Following these procedures, he gave up athletics and re-registered as male. Because of Weston’s profile as an Olympic athlete, he received media attention when the story became known in 1936. The 1930s saw an increase in reports of ‘sex change’ in the British popular press (Oram, 2011), and there was a series of articles on Weston between 1936 and 1938 (*Daily Mirror*, 1936, 1938; Heggie, 2010; *News of the World*, 1936; Wickets, 1937). The headline of the *News of the World* article – ‘“Girl” athlete’s new life after change of sex’ – is representative of the fact that, while there is no evidence that Broster performed any surgeries on trans individuals (King, 2002) these surgeries were often presented using the language of *changing* sex.

In his own account, Broster described how Weston was admitted to Charing Cross in 1935 for ‘an opinion on his sex’ (Broster et al., 1938: 47). Broster comments on Weston competing in the Olympic games ‘as a girl’ and states that following this, while training in massage, he learned anatomy and ‘became conscious of the fact that he was abnormal and that he became attracted to girls – in particular one’. Upon examination, it was discovered that he had a hypospadiac penis, a ‘cleft vagina-like scrotum’ and two small undescended testes. Weston was admitted to the male ward and a number of plastic operations were carried out. His description of Weston is markedly different to his example of the woman who ‘adopted a son!’:

This man succeeded in attaining male sexuality against every disadvantage. He is a triumph of instinctual development … In his personality, his psychosexual life and in every way he was a complete male – it was only the misfortune of his environment which prevented him showing it. (Broster et al., 1938: 48)

While Broster did not describe Weston’s atypical sex anatomy as subtracting anything from his masculinity, it is instructive to see this passage in comparison with the previously discussed case. (Heterosexual) maleness is an achievement, associated with agency and success. Femaleness, however, is associated with passivity, lack and failure; the female body is the situation that Weston remarkably overcomes.

## The 1940s and Michael Dillon

The ‘sex change’ narrative that structured popular understandings of Weston was popular in the press in the 1930s, and this coincided with new medical claims and published accounts of surgical procedures to ‘manage’ atypical sex anatomies (Oram, 2011). In effect, the networks of ‘sex change’ were expanding to incorporate heterogenous actors, not limited to communities of medical professional practice (Latour, 1993). In the 1940s, press attention continued to present Broster’s work to a wider public (*News of the World*, 1943). Following the publication of these stories, medical professionals received requests from individuals who found a language in which to express their feelings of having been raised as the wrong sex. Sexologist Norman Haire claimed in 1950 that the news reports would always be followed by requests to medical professionals for what they considered to be medical interventions that would help them make their biological sex fit their personal sense of a ‘correct’ sex (Haire, 1950; King, 1995). Despite the fact that many of the press reports were about intersex surgeries, this reporting provided a medical language for individuals who wanted to access the medical community to meet their needs for surgery. Simultaneously this press coverage obscured or even erased the existence of intersex as a category separate from trans. As a consequence, doctors were keen to redraw the boundaries between these two categories.

In 1940, Clifford Allen argued that treatments should be strictly divided between psychotherapeutic treatment for individuals who do not have an identifiable atypical anatomy and surgical intervention for individuals who do (Allen, 1940). Broster had the authority to decide or deny physical treatments, or to refer to Allen, who had the authority to decide or deny psychological treatments. In Allen’s chapter of *The Adrenal Cortex and Intersexuality*, he described a case study of a woman who ‘wants to be a man’ so that she could marry her girlfriend. No physical abnormality was found, so Allen offered psychotherapy to address her (in his view) homosexuality, which she refused (Broster et al., 1938: 106–107). Despite the agency that Broster narrated in his case studies, medicine seems to have remained the authority and conveyer of legitimacy for individuals who did not conform to biological or psychological norms of sex.

Broster’s 1944 book, *Endocrine Man*, sharpens the distinction between individuals with what the medical profession defined as physical or psychological abnormalities. He was very clear that in both cases, treatment of some kind should be pursued, to obtain as close a state to ‘normal’ as possible:

When their troubles are due to natural causes their plight is pitiable. Society in general is not a respecter of persons, is suspicious, and indeed often hostile to these abnormals. This attitude should be rightly reserved for the decadent imitators and propagandists of these perverted states, who form a festering sore in our midst. (Broster, 1944: 95)

Broster was confident of the ability of medical intervention (whether surgical, hormonal, or psychotherapeutic) to create and maintain a dichotomous, two-sex model of sex and sexuality. The language that Broster used demonstrates a moral difference between what the medical profession considered biological and psychological abnormalities. When biological ‘abnormalities’ were apparent, the individual was deserving of pity. However, when the medical professional identified psychological ‘abnormalities’, there was a moral and social obligation on the individual to submit to treatment (possibly psychotherapeutic, possibly surgical). Those who refused treatment for the medical profession’s definition of psychological and sexual abnormality were considered propagandists worthy of scorn and hostility.

Individuals who wanted access to ‘sex change’ surgeries had to navigate a medical terrain structured by these moral distinctions. Michael Dillon, registered female at birth in 1915, had a mastectomy in 1942. Later that year he made contact with world-leading plastic surgeon Sir Harold Gillies about possible further surgery. Dillon amended his birth certificate to male in 1944; at the time, this was possible with a medical certificate (acquired from a doctor John Cooper, in Bath) and a certificate from a member of the family (signed by his cousin, Maude Eileen Beauchamp). After re-registration, he underwent a series of treatments including what is generally considered to be the world’s first phalloplasty from 1946 until 1955 (Hodgkinson, 2015; Kennedy, 2007). While Dillon has been discussed in trans histories, and it is not clear whether he had any anatomical features that could be described as intersex variations, his story nonetheless informs scholarship on intersex in a number of important ways. Gillies recorded a diagnosis of hypospadias upon admittance, even though the medical notes make explicit reference to female genitalia (Transgender individuals: Laurence Michael Dillon, n.d.). Dillon was described as a ‘hermaphrodite’ in one of Gillies’ letters sent to enlist other medical professionals in this untried and controversial treatment; in another, Gillies included a letter from Dillon recounting an adventure in the merchant navy, saying ‘if you would care to read this letter from him you will see what kind of active life he has made for himself. He is a real tough boy’ (Transgender individuals: Laurence Michael Dillon, n.d.).

In sum, Dillon was actively navigating a medical encounter structured by Gillies’ considerable authority (his letters are all addressed to ‘Sir Harold’ despite the increase in affection in Gillies’ responses, developing from ‘Dear Dillon’ in 1946, to ‘Dear Michael’ in 1947, and ‘My Dear Michael’ by 1954). Although Dillon was excluded from the community of professional medical practice, he was an actor who had to navigate this network (Star, 1990). Gillies also had to navigate the complex field of ‘sex-change’ controversy. It seems there was a tacit agreement between them to use the logic and language of intersex, rather than the more controversial topic of ‘sex-change’ (Transgender individuals: Laurence Michael Dillon, n.d.). For Dillon, this involved getting a medical certificate from an independent medical professional (John Cooper) and making the change to his birth certificate, as well as presenting himself in particular ways through his correspondence. Gillies’ recording of ‘hypospadias’ on the medical notes can also be read as an attempt to avoid the controversy of sex-change through recourse to the language of known ‘abnormalities’ in sex characteristics.

Dillon’s experience with Gillies was certainly not typical of individuals who approached the medical profession looking for what was referred to as ‘sex change’ at the time. While he successfully obtained the surgical and hormonal interventions he wanted, it is clear that other individuals did not find this negotiation easy, or even possible. In 1946, Dillon published *Self: A Study in Ethics and Endocrinology*, in which he argued that when there is ‘an incompatibility between the mind and the body, either the body must be made to fit the mind … or the mind be made to fit the body; *and that is for the patient himself to judge if he be of age*’ (1946: 65, emphasis added). Dillon’s opinion was not widely shared in the medical community. For Broster and Allen, the incompatibility between mind and body, if unaccompanied by intersex variations, should be treated by making the mind fit the body. A moral hierarchy was defined, whereby ‘natural’ or biological differences from a norm deserved pity, and other differences deserved contempt. Significantly, Dillon’s book in 1946 challenged who got to decide what and when treatment is appropriate, suggesting that legitimacy could come from self-determination, not medical authority.

## Roberta Cowell

Similar to Broster and Allen’s cases, there were differences in the experience of men and women approaching the medical profession for surgery. Roberta Cowell was born in 1918 and assigned male. In her 1954 autobiography she stated: ‘Since May 18th, 1951, I have been Roberta Cowell, female. I have become woman physically, psychologically, glandularly and legally’ (Cowell, 1954: 5). Cowell’s account of herself has been considered in a number of histories of trans politics and experiences (e.g. Hausman, 1995; Meyerowitz, 2002). However, in her autobiography she invoked the language of intersex, specifically through the contemporary language of ‘hermaphroditism’:

how extensive the hermaphroditism was could not be decided without a more detailed examination and laboratory tests. There was a possibility that some of the internal organs might be female … This knowledge raised my morale very considerably. The intense shame I had felt began to disappear. Once I realised that my femininity had a physical basis I did not despise myself so much. I now knew, of course, that I was physically abnormal, but I could accept a degree of involuntary femininity without losing self-respect. (Cowell, 1954: 42)

Cowell reinforced this intersex narrative throughout her biography, and described her transformation as one that was happening anyway, and that medical intervention helped along. She maintained this narrative, even in her final published interview in 1972:

I was a freak. I had an operation and I’m not a freak any more. I had female chromosome make-up, XX. The people who have followed me have often been those with male chromosomes, XY. So they’ve been normal people who’ve turned themselves into freaks by means of the operation. (*Sunday Times*, 1972)

This statement conforms to the moral hierarchy described earlier. As a biological and ‘natural’ issue, Cowell can frame her narrative as one that deserves pity, rather than contempt. Cowell is attempting to gain legitimacy through this use of the intersex narrative. However, this framing of legitimacy is deeply problematic. Individuals with intersex variations were (and continue to be) subject to experimental medical treatments, often without consent or disclosure; they did not have unproblematic access to legitimacy. Cowell’s narrative is also potentially divisive and damaging to trans politics, as it suggests that biology and medicine are the correct arbiters of legitimacy and frames trans people as ‘freaks’ in the process. Cowell positions herself as more legitimate and deserving of medical attention, which establishes a moral hierarchy of legitimate and illegitimate trans subjects. This is a significantly different approach to Dillon, who appealed for legitimacy based on self-determination, rather than medical authority.

In Cowell’s autobiography, she talks about meeting Dillon, but only briefly. Liz Hodgkinson’s biography of Dillon, however, suggests a long and intense relationship with Dillon introducing Cowell to Gillies, giving advice on how to negotiate Gillies’ authority, and writing to Gillies himself stressing the very heterosexual relationship between himself and Cowell (Hodgkinson, 2015). Cowell’s autobiography is vague about her medical treatment, but from Dillon’s biography it appears that she was not able to obtain an orchidectomy, so as to gain a medical affidavit saying she had been wrongly registered male at birth. Dillon had offered to perform the operation if she was unable to find anyone else to do the procedure and in 1950, Cowell signed a document absolving Dillon of responsibility if anything went wrong. He performed the operation in secret and in 1951, Cowell reregistered as a woman.

In Cowell’s self-description, she clearly (and understandably) invoked the logic expressed by Broster and Allen. Intersex treatment seemed to involve a consultative process that (at least to some extent) took into account an individual’s ‘psychology’, social role, and ‘self’, something that was not true of contemporary treatment of individuals requesting ‘sex change surgeries’. To the medical profession, ‘transsexuals’ were seen as threatening, described as ‘decadent imitators’ deserving of hostility (Broster, 1944: 95). In contrast, intersex individuals were figured as innocent exceptions in ‘need’ of medical intervention. Thus, to individuals like Cowell, it was preferable to appropriate an intersex narrative. Within this narrative, surgery is presented as helping a process that is already under way: the discovery and affirmation of a ‘true sex’. In the 1940s, these procedures were generally for adults. However, treatments were happening at an earlier and earlier age, often without consent or disclosure after the fact. This allowed medical professionals to take the ‘self’ of their patients into account less and less, while still maintaining a strict delineation between biological and psychological ‘abnormalities’, and the moral hierarchy that accompanied their treatments.

## The 1950s and the intersex child

In the late 1940s a new speciality – paediatric urology – emerged. In his political history of the NHS, Charles Webster (2002) argues that prior to the Second World War, the provision of health services in Britain were ‘haphazard’. After the war, there was a drive to formalize, standardize, and to create a National Health Service that was world leading. David Innes Williams, resident to St Peter’s Hospital for Stone in London in 1948,^6^ and soon after urological registrar to Great Ormond Street Hospital, was described as the ‘father of paediatric urology’ in the UK (Woodhouse, 2015). In an interview in 2011, Williams claimed that, unlike other specialities, urology had not gained much from the war (Hodgson, 2011). With the inauguration of the NHS in Britain in 1948, however, paediatrics began to flourish. Williams recounts how in the early years of the NHS his senior paediatric registrar colleagues at Great Ormond Street ‘went out and got a job in the South of England – they sent me back their stuff, so suddenly there was a lot of material’ (Hodgson, 2011: 53).

‘Material’ for a paediatric urologist included case notes, but also actual children. The newly established NHS enabled networks of practice in which people (doctors, patients and families) and things (including case notes) were drawn together (Latour, 1993). Williams narrates a story of a child admitted in 1948 to St Peter’s Hospital while Williams was there as a registrar in urology. Williams recounts that none of his senior colleagues knew how to treat the child, because of a lack of specialization in paediatric urology in the UK at the time (*The Telegraph*, 2013). Williams also claims a poor standard in the medical textbooks that were available in this area:

There was a text book on Urology published by Winsbury-White in ‘48 while I was a resident there [St Peter’s], and it is an extraordinary thing, more than half of the contributors are retired, it is so old fashioned … so it was all going to change, that generation was going mostly. (Hodgson, 2011: 52)

Thus, after Williams moved to Great Ormond’s Street, he published *The Urology of Childhood* (Higgins et al., 1951). The book was organized into 16 chapters, the final three of which were ‘Congenital External Anomalies’, ‘Diseases of the External Genitalia’ and ‘Hermaphroditism and Disorders of Sexual Development’. *The Urology of Childhood* has a focus on external genitalia rather than glands, due to the disciplinary differences between urology and endocrinology, although he makes oblique reference to adrenal treatments not being effective (249–250). Williams did not explicitly reference Broster or Allen. When he did reference an endocrinologist, it was GIM Swyer, whose ‘hitherto undescribed form’ of ‘male pseudohermaphroditism’ in 1955 would go on to be known as ‘Swyer syndrome’ (Swyer, 1955).

Although Broster and Allen were not recalled in this new paradigm, psychology was invoked in this book. When discussing hypospadias, Williams states:

It is generally recognized that restoration of normal masculine micturition must, if possible, be achieved before the school age, say 5 to 8 years. For psychological reasons it is obviously important that the boy at school should pass urine like his fellows. (Higgins et al., 1951: 219)

Unlike Broster and Allen’s focus on adult sexuality, here it is the *child’s* psychology that is in question. In the case of hypospadias, surgery is invoked as enabling standing-up urination, which is ‘obviously important’ for healthy male development. Standing-up urination is historically important for a person’s place as a man in society (Mak, 2005: 28–9). However, for Williams here, it is not the social position of the urinating individual that is important, but the psychological well-being that comes with the successful public performance of standing-up urination. And surgery is suggested as the tool to enable this psychological development. In what he describes here as ‘hermaphroditism’ or ‘disorders of sexual development’, there is a similar suggestion: ‘The guiding principle in the treatment of all this group of cases must be adaptation of the external appearance to the psychology of the child rather than to the histology of the gonad’ (Higgins et al., 1951: 249). Medical professionals were constructing standing-up urination as a new component of what a ‘normal’ child was.^7^ Psychological development is invoked as both simple (divided into male and female types, which are easily identified by a urologist) and precarious (easily threatened by urinating sitting down, and in need of surgical intervention).

The following year, Williams published ‘The Diagnosis of Intersex’ in which he again emphasized the lack of knowledge and uncertainty in this field:

The problem of the infant of doubtful sex is one which occasionally faces every clinician who concerns himself with children’s work, and yet, in spite of the importance of a correct decision, it is still impossible to obtain guidance of this matter from the ordinary textbooks. Too often the decision is postponed to see which way the child will develop; the parents are left without guidance, and their natural anxiety is communicated to the child, who is thus in danger of becoming a psychological as well as a surgical problem. (Williams, 1952)

The precariousness of childhood psychological development is again invoked as a justification for early intervention. Interestingly, parental anxiety is also invoked as a threat to psychological development, and one which surgery on the child can manage. The article demonstrates the prevalence of ‘doubtful sex’, locates it as a childhood issue, emphasizes the importance of swift and surgical response, and depends upon a strict delineation between male and female bodies.

## Mid-1950s: Diagnosing sex in the UK before John Money

CN Armstrong, endocrinologist at the Royal Victoria Infirmary in Newcastle, was a prominent expert in intersex (which he described as ‘diversities of sex’ in which he included homosexuality as well as ‘hermaphroditism’) in the 1950s.^8^ Armstrong was concerned with the ambiguities posed to both medicine and the law by clinical experience with intersex variations: ‘So far the law has never defined sex, which is extraordinary in view of the homosexual laws in this country, and it is sometimes very difficult to say to which sex an individual belongs’ (Armstrong, 1955: 1176). For Armstrong, the existence of variations in sex anatomy makes the boundary between male and female bodies uncertain. Consequently, if biological sex is not easy to define, then this uncertainty is increased in attempting to define homosexuality; and as homosexuality was at the time defined as a problem (whether as crime or disease), this provokes uncertainty into how to deal with this problem. Heterosexuality was a sign of clinical success, even if clinicians and other medical professionals did not share a definition of homosexuality, or even heterosexuality; in fact, their definitions could be very much in conflict with one another (Mak, 2015). In terms of sexuality, medicine is portrayed as in service to the law, which is argued to be on shaky foundations when it comes to defining sex and sexuality.

In 1952, Armstrong had suggested that the legal sex should be defined solely on gonads. In the discussion of a 17-year-old female patient with internal testes or ‘male pseudohermaphroditism’, Armstrong stated:

The legal determining factor in intersex cases is the anatomical structure of the gonad independent of the social or sexual inclinations or external appearance of the subject. In this case, therefore, the legal sex is male, and although one would have no hesitation in defending this patient's desire to continue living as a female, the clinical problem does arise as to how far a surgeon is justified in carrying out a plastic operation to make a vagina which, in this case, has already been requested. (Armstrong, 1952: 302)

While Armstrong saw no reason to force the patient to make their social sex match their gonadal sex, he does question the practice of surgically making the body fit the social sex, and thereby allowing female patients (gonadally and legally defined as male) to have sexual relationships with other men. While there was a consultative process of sorts beyond the ‘truth’ of the gonads, only that which was normative in terms of ‘social sex’ could be sanctioned in adults.

Surgery seems to have fulfilled several social roles here, including supporting vague ideas of psychological well-being. In the UK in the 1930s and 1940s, medical professionals like Broster and Allen had separated the psychological treatment of transsexuals from the surgical and hormonal treatment of intersex individuals. In the early 1950s, Williams further suggested that surgery could be put in the service of ‘healthy’ psychological development. This seems to suggest a John Money-like approach to surgery, as a tool to construct and maintain social and cultural norms for the psychological well-being of individuals, in the UK, in the years preceding Money’s influence. However, Williams’ notion of ‘psychology’ was ill defined, while Money’s publications sought to present methods and guidelines that were apparently backed by empirical data and theoretical justifications. Also, Money used surgery *as* child psychology, whereas for Williams, surgery should be used to make the body of the child fit their pre-existing psychology (however vaguely defined). Money had access to advanced discourses of psychology and other emerging fields. In fact, as Morland (2015) argued, Money’s theories were indebted to his (mis)reading of the contemporary American field of cybernetics. Williams’ approach was different, embedding intersex ‘management’ within the context of a newly developed NHS, which entitled individuals to certain treatments and care ‘from cradle to grave’ (Rivett, 1998). The National Insurance Act (1946), National Health Service Act (1946), along with the Children Act (1948) and the Universal Declaration of Children’s Rights (1948), constructed children as deserving of rights, and of medical treatments, while also still positioned within certain norms of the family. The NHS solidified ideas about medicine being in the service of the state. For Williams, surgical interventions functioned to stop variations of biological sex becoming a ‘psychological problem’, and were therefore in the service of securing the parent–child relationship from anxiety, and thus, within the context of welfare, also in service of the state.

## Conclusion

The UK-specific context between 1930 and 1955 illuminates discussions of Money’s significance to the history of intersex; it also has significance for scholarship on intersex more broadly. The pre-Money era in intersex medical ‘management’ in Britain was a complex terrain of shifting medical, legal and popular discourses. In *Hermaphrodites and the Medical Invention of Sex*, historian Alice Dreger described the period at the end of the 19th and start of the 20th century as the ‘age of the gonads’, when sex was theoretically defined by the existence of ovaries or testes. In practice, however, medical professionals did not rigidly attempt to make the lived or social sex match up to the ‘truth’ of the gonads (1998: 158). Geertje Mak has critically elaborated upon Dreger’s thesis to suggest that from the early 1900s, physicians also began to take the ‘self’ of individuals with atypical sex anatomies into consideration (Mak, 2005, 2012). Mak argued that medical encounters were more complex than submission to medical authority and the ‘truth’ of the gonads, and often took into account social roles and the individual’s sense of self, alongside physical evidence.

Evidence from Broster and Allen’s case studies supports Mak’s argument that ‘the age of the gonads’ needs further clarification and critical elaboration. Broster was performing routine intersex surgeries in the UK in the 1930s, claiming in 1938 that he had seen hundreds of patients. While there was a sense that glands and hormones were determinative of some sense of a ‘true sex’, in clinical practice, this was inseparable from cultural norms about men and women, social roles, and developing ideas about psychology. Broster was fascinated by the psychology of his adult patients, despite his disciplinary focus on gonads. Broster’s case studies support the argument that ‘psychology’ and the self were taken into account alongside evidence of gonads in adult patients. However, decisions were still made within strict norms of gender and sexuality. While ‘true sex’ was still held to exist *somewhere*, social norms as they pertained to women and men’s bodies structured adult clinical encounters.

This history intersects with a popular press in Britain that had, since the 1930s, presented intersex surgeries to the public in the language of ‘sex-change’, as well as with a history of individuals seeking out surgery to make their body better fit what would come to be known as their gender identity. These individuals blurred the boundaries between the medical and non-medical, and between the biological and the psychological, as well as the seemingly impermeable barrier between the hierarchized binary relationship between male and female bodies. Dillon and Cowell problematized these boundaries in different ways, which led to a need within the medical profession to purify, redraw and maintain strict boundaries, including between trans and intersex. Broster and Allen’s approach was that intersex was a biological condition demanding pity and medical intervention, while trans was a psychological condition that should prompt contempt and psychotherapeutic intervention. This has relevance for contemporary trans and intersex politics. Both trans and intersex are still pathologized within a medical setting, but in different ways. Medical approaches to trans and intersex are still differently organized; medical interventions for trans individuals are approached with a caution that is not replicated in intersex interventions:

Even after years of criticism from intersex people, many providers are quick to perform surgery on bodies of babies and young children that they consider abnormal … At the same time, they hesitate to act in cases where trans individuals request surgery. (Davis et al., 2016: 491)

The moral dichotomy evidenced by Broster and Allen also continues in contemporary intersex negotiations with medical authority. With the introduction of the term ‘disorders of sex development’ or ‘DSD’, individuals find themselves in a position where adopting or refusing the label of ‘disordered’ affords access to different treatments, experiences of treatments, feelings of belonging to a community, and self-legitimacy (Davis, 2015).

By the early 1950s, Williams claimed that intersex was something that all clinicians who work with children would encounter. With the development of paediatric urology and the formalization and standardization of medical care in the NHS, intersex became an issue of childhood, and the imperative to ‘fix’ or normalize children to one of two sexes through surgery was framed as a humanitarian issue in the service of the child’s psychological development, and as an intervention that should happen sooner rather than later. There are differences in Williams’ approach to that which would come from Money and colleagues in the USA: psychology and surgery were invoked in different ways. Eder (2011) has argued that Money’s work did not necessarily represent a ‘radical departure’, but a formalizing of existing procedures and discourses in the USA. There are certainly distinctly British ways of thinking about children during this period. However, it is significant that three years prior to Money’s influential 1955 publications, a textbook of paediatric urology in Britain was recommending early surgery for psychological reasons, as well as to reduce parental anxieties – issues that still affect the intersex community and which are the focus of intersex activism.

The shift of focus onto the child naturalized the redrawn medical boundaries between trans and intersex, between biology and psychology, and between the child and the adult. John Money has merited more detailed study than any other author in this field (Downing et al., 2015). To some extent, Money’s work confirmed and supported shifts in approaches and clinical practices that were already in motion. However, notions of psychology and the role of surgery were invoked differently in the UK. Williams agreed that ‘doubtful sex’ represented an obviously surgical problem, and one that must be treated early, so as to avoid it becoming a psychological problem. However, for Williams, surgery should modify the body to fit the child’s existing psychological sex, whereas Money’s approach used surgery *as* child psychology to produce and maintain coherent sex and gender. Boundaries between the child and the adult are still significant for intersex, as emphasized by the publication in 2010 of a set of ethical principles and medical recommendations by the ‘Bioethics and Intersex’ working group within the German Network DSD/Intersex (Wiesmann et al., 2010). This paper constructs the child as both an individual with ethical needs, as well as within a family with complex rights and responsibility. Crucially however, the child is also constructed as a *future adult*, and thus any medical intervention needs to grapple with the ethics of the well-being and the rights of both the child and the future adult. These are fuzzy and heterogenous overlapping categories, despite the purification attempts of the medical profession.

Focusing on the UK in the period before Money’s guidelines would become paradigm offers a historical view of the tensions and intersections of trans and intersex politics, experiences and representations, tensions and intersections that continue into the present. Trans histories are relevant but not identical to intersex history. The histories intersect, but are divided by the moral dichotomy described in this article, as well as very different experiences of the medical profession.^9^ However, as Alice Dreger and April Herndon argue, ‘even though there may be differences between intersex and trans-gender, there are also reasons for intersex and trans activists to unite’, particularly around human rights (Dreger and Herndon, 2009: 213). While this is true, intersex people have been denied full human rights in a very specific way. Individuals and organizations have had to fight for intersex issues to be considered human rights issues, often through a framework of bodily integrity. A 2015 editorial in the *BMJ* noted the relevance of the European Union Agency for Fundamental Rights April 2015 recommendation that member states avoid non-consensual medical treatments on intersex individuals (Liao et al., 2015). Despite this recommendation, the article stated that UK practice is still to ‘manage’ atypical sex anatomies with genital surgery. Recognition of intersex rights in the UK seems to be at a much earlier stage than trans rights. In the UK, in January 2016, the House of Commons’ Women and Equalities Committee published a report on transgender equality which gave recommendations considered positive by a number of trans groups and communities (House of Commons Women and Equalities Committee, 2016). While intersex groups provided significant evidence to the committee, intersex was only mentioned briefly, in terms of an imperative for the government to consider how best to address the needs of intersex children and adults at an unspecified time in the future (Centre for Law and Social Justice, 2015). It seems that there is an endless deferral of giving intersex its due focus. The particularities of intersex politics in the UK demands a specific focus on intersex history in the UK, even if this history cannot be disentangled from those of trans individuals, technologies and politics.

## Notes

1 Variations in biological sex characteristics have been variously described as ‘hermaphroditism’, ‘intersex’, and more recently as ‘disorders (or divergence/differences) of sex development (DSD)’. See Reis, 2007 and Davis, 2011.

2 The language around procedures and identities for individuals who do not identify with the sex they were assigned at birth has shifted considerably since the period covered in this article. As far as is possible, I will use the terms that were available to the medical professionals that I explore, including the emerging term in this period: ‘transsexuality’. When making more general statements or linking to contemporary politics I will use ‘trans’, to refer to a person ‘who feels that the sex or gender they were assigned at birth does not match or sit easily with their sense of self (their self-identified gender)’ (Gendered Intelligence: Trans Identities, 2016).

3 Throughout the article I will use pronouns used by individuals *after* legal re-registration or medical intervention, so male for Weston and Dillon and female for Cowell.

4 Allen’s views became more Freudian during his career (see Allen, 1940, 1962, 1966).

5 Hypospadias is the medical term that refers to a variation of the positioning of the urethral meatus from the tip of the penis. Hypospadias can vary in severity and estimations of incidence range from 1 in 150 to 1 in 300 male births. However, research has suggested that the variation in the position of the urethral meatus in the general population is much higher than imagined and that urological expectations for ‘normality’ in penis morphology may be stricter than the general population (Fichtner et al., 1995).

6 This hospital closed in 1992, and services moved to Middlesex Hospital; Middlesex Hospital closed in 2005, with services then moving to University College Hospital London (St Peter’s Hospital for Stone London, 2016).

7 For more on the co-construction of ‘normal’ children and the discipline and practice of psychology see Rose, 1990 and Burman, 2010.

8 He was a founding member of the section of endocrinology at the Royal Society of Medicine in 1946 and later its president (Munks Roll Details for Charles Nathaniel Armstrong, 2016).

9 For contemporary intersex narratives including experiences of medicine (mainly in the USA), see Davis and Feder, 2015.
